# The forbidden zone for sleep is more robust in adolescents compared to adults

**DOI:** 10.3389/frsle.2023.1304647

**Published:** 2024-01-04

**Authors:** Allison J. Monterastelli, John Adams, Charmane I. Eastman, Stephanie J. Crowley

**Affiliations:** ^1^Biological Rhythms Research Laboratory, Department of Psychiatry and Behavioral Sciences, Rush University Medical Center, Chicago, IL, United States; ^2^Department of Behavioral Sciences, Olivet Nazarene University, Bourbonnais, IL, United States

**Keywords:** wake-maintenance zone, circadian amplitude, sleep, evening alertness, sleep propensity, puberty, adolescence

## Abstract

**Introduction:**

The propensity for sleep shifts later as puberty progresses. The present analysis examines whether the circadian-dependent wake maintenance zone, or forbidden zone for sleep observed in the evening just before habitual bedtime is more pronounced in late to post-pubertal adolescents compared to adults and may partly explain late sleep onset in maturing adolescents.

**Methods:**

Forty four healthy late/post-pubertal adolescents (aged 14.3–17.8 years, 23 female) and 44 healthy adults (aged 30.8–45.8 years, 21 female) participated in an ultradian light/dark protocol for 3 days cycling between 2-h wake periods (~20 lux) and 2-h nap periods (~0 lux) without external time cues. The dim light melatonin onset (DLMO), a measure of circadian phase, was measured immediately before the ultradian protocol by sampling saliva every 30 min in dim light. Wrist actigraphs were used to assess sleep onset latency and total sleep time during the naps that occurred during the ultradian sleep/wake schedule. Sleep episodes were grouped into 2-h bins relative to individual DLMOs (28–56 naps/bin). Sleep onset and total sleep time were compared between adolescents and adults as well as between males and females within each age group.

**Results:**

Adolescents took significantly longer to fall asleep compared to adults during naps that occurred in the 4 h window surrounding the DLMO [2h before DLMO *t*_(50)_ = 2.13, *p* = 0.04; 2 h after DLMO *t*_(33)_ = 3.25, *p* = 0.003]. Adolescents also slept significantly less than adults during naps that occurred in the 4-h window surrounding DLMO [2 h before DLMO t_(51)_ = −2.91, *p* = 0.01; 2 h after DLMO *t*_(33)_ = −1.99, *p* = 0.05]. Adolescent males slept less than adolescent females in naps that occurred in the 2 h window after the DLMO [*t*_(14)_ = −2.24, *p* = 0.04].

**Discussion:**

Compared to adults, late/post-pubertal adolescents showed greater difficulty falling asleep and maintaining sleep around the time of their DLMO, which usually occurs a few hours before habitual sleep onset. A greater amplitude in the circadian-driven forbidden zone for sleep could be an additional physiological mechanism explaining why maturing adolescents find it difficult to fall asleep early, increasing the risk for restricted sleep in the context of early school start times.

## Introduction

A well-documented change during adolescence is the significant shift in habitual sleep patterns to a later clock time than earlier developmental stages (Carskadon et al., 1993; Roenneberg et al., 2004; Crowley et al., 2014; Maslowsky and Ozer, 2014; Fischer et al., 2017). Delayed bedtimes are partially explained by an increase in nightly societal pressures including a rise in social demands with new access to social media and increased responsibilities associated with educational demands and part- time work (Wahlstrom et al., 2017; Scott et al., 2019). Nightly sleep opportunity for adolescents in middle school and high school is bookended with early school start times (Crowley et al., 2014; Bowers and Moyer, 2017; Tarokh et al., 2019), resulting in a truncated sleep opportunity for many adolescents during the school week. Despite the current recommendation for adolescents to sleep 8–10 h per night (Hirshkowitz et al., 2015; Paruthi et al., 2016), < 8% of high school students report sleeping 9 hours per night, and nearly 70% of students report sleeping 7 or less hours per school night (Wheaton et al., 2016). This amount of sleep loss can pose substantial risk to development as too little sleep in teens is associated with increased incidence of weight gain, obesity, mental illness, drowsy driving, high risk behavior, poor academic outcomes and a slew of other risk factors pertaining to physiological, mental, and overall wellbeing (Pasch et al., 2010; McKnight-Eily et al., 2011; Arora and Taheri, 2014; Simon et al., 2019; Alfonsi et al., 2020; Lin et al., 2020). Late bedtimes are not only caused by increasing social pressures, however, and are also due to physiological changes during puberty that shift the propensity for sleep to a later time of day (Carskadon, 2011; Crowley et al., 2018).

The Two Process Model of Sleep Regulation first described by Borbély (1982) has been a valuable tool to understanding sleep behavior during development (Carskadon and Acebo, 2002; Carskadon et al., 2004; Jenni and LeBourgeois, 2006). According to this model, two processes contribute to the regulation of sleep: the Homeostatic Sleep-Wake System (Process S) and the Circadian Timing System (Process C). Process S dictates that the propensity for sleep increases over the course of a waking day and this pressure dissipates with sleep. Simultaneously, the propensity for sleep is controlled by Process C – the ~24-h endogenous central circadian clock—which has been localized to the superchiasmatic nuclei (SCN) of the hypothalamus and is largely independent of prior sleep and wake. When these two processes are cast in opposition of one another and are ideally aligned in time, Process C maintains sleep at the end of the night as Process S dissipates and maintains alertness at the end of the waking day as sleep propensity from Process S accumulates. Theoretically, the interaction between Process S and Process C across 24 h allows for consolidated wake and consolidated sleep (Dijk and Czeisler, 1994).

The dynamics of the two processes regulating sleep and wake change during puberty in a way that favors greater physiologic alertness in the evening in mature adolescents. Adolescents' central circadian clock marked by the dim light melatonin onset (DLMO) shifts later with maturation despite the same sleep/dark history as pre-pubertal adolescents (Carskadon et al., 1997). A circadian phase delay of activity rhythms is also observed in other post-pubertal mammals (Hagenauer et al., 2009; Melo et al., 2016), confirming a biological underpinning to the delayed 24-h rhythm of sleep propensity. To probe Process S, Taylor et al. (2005) measured speed of falling asleep (sleep onset latency) in prepubertal (Tanner 1; *n* = 9) and post-pubertal (Tanner 5; *n* = 11) adolescents every 2 h across 36 h of extended wake in a constant routine protocol. Post-pubertal adolescents took longer to fall asleep in the 4 h after scheduled bedtime (22:00) compared to pre- pubertal adolescents. These data, along with models of slow wave activity accumulation in pre- and post-pubertal adolescents by Jenni et al. (2005), suggests a slowing of sleep pressure accumulation across waking in post-pubertal adolescents. These data have been interpreted as Process S becoming more permissive of later bedtimes as adolescents mature.

The circadian-dependent alerting at the end of the waking day, however, may also makes it more difficult to fall asleep in the few hours before habitual bedtime. This time of day preceding habitual bedtime is called the “wake-maintenance zone” or “forbidden zone for sleep,” a phenomenon first described by Lavie (1986). In his study, adult men were asked to resist or attempt sleep during an ultra-short nap protocol (7 min dark/sleep alternated with 13 min light/wake) across 36 h. Regardless of whether participants were instructed to resist or attempt sleep, they displayed a decreased ability to sleep in the 2–3 h preceding habitual bedtime and this was followed by an abrupt increase in sleep propensity around habitual bedtime (the “sleep gate”). The forbidden zone for sleep has since been researched further by a number of groups (Folkard and Barton, 1993; Lack and Lushington, 1996; Eastman et al., 2017; McMahon et al., 2018; Zeeuw et al., 2018), but has not been adequately described in adolescents. In an attempt to further examine the underlying sleep-wake mechanisms that drive delayed sleep propensity of mature adolescents, we aim to examine sleep propensity controlled by the circadian timing system during the so-called “forbidden zone for sleep” while attempting to minimize Process S using a 4-h ultradian light/dark cycle in late to post-pubertal adolescents aged 14–17 years and compare them to adults aged 30–45 years. Based on prior work documenting enhanced evening alertness in mature adolescents, we hypothesize that adolescents will display more difficulty sleeping in the hours before habitual bedtime compared to adults.

## Materials and methods

### Participants

This study was conducted at the Biological Rhythms Research Lab at Rush University Medical Center in Chicago, Illinois from 2012 to 2016. The study enrolled 44 healthy adolescents (16.2 ± 1.0, 23 female at birth) and 44 healthy adults (38.0 ± 4.2, 21 female at birth) from the Chicagoland area. Participants self-identified their race as African American (19 adolescents and 15 adults), White (17 adolescents and 26 adults) or another race (8 adolescents and 3 adults). Thirty-four adolescents identified as non-Hispanic, and 39 adult participants identified as non-Hispanic. Race and ethnicity for adolescent participants were confirmed by a parent or guardian.

Participants were medication free, aside from female participants who used hormonal contraception (*n* = 2 adolescents, *n* = 2 adults). Participants reported caffeine intake < 300 mg/day. Participants did not endorse a history of neurological disorders, psychotic disorders, bipolar disorders, psychopathology, metabolic disorders, infectious illness, sleep disorders, chronic medical conditions, physical disabilities, or developmental delays. Participants did not display symptoms of depressed mood (CES- D ≤ 16) (Radloff, 1977). Participants were not color blind or color deficient [Ishihara Colorblindness Test (Ishihara, 1972)] and did not endorse a history of eye surgery. Body Mass Index (BMI, kg/m^2^) was calculated for adolescents using the Center for Disease Control's public tool (https://www.cdc.gov/healthyweight/bmi/calculator.html). Controlling for age and sex, the BMIs for adolescents ranged from the 6th to 97th percentiles (16.5–32.1 kg/m^2^). BMI for adult participants ranged from 17.3 to 34.9 kg/m^2^.

Pre-study sleep habits were assessed using sleep logs and questionnaires. Eligible participants reported sleeping between 6 and 10 h per night with wake times between 06:00 and 11:00. The Munich Chronotype Questionnaire (MCTQ) was used to assess free-day mid-sleep times (Roenneberg et al., 2004). On average, the free-day mid-sleep times were 04:10 ± 1:12 for adolescents and 04:14 ± 1:12 for adults. The Morningness Questionnaire of Smith and colleagues was used to assess preferred time of day (circadian phase preference) (Smith et al., 1989). Scores can range from 13 to 55. Scores below 22 indicate evening preference, scores above 44 indicate morning preference (*n* = 3 adolescents; *n* = 13 adults), and scores between 22 and 44 indicate a preference for neither morning nor evening (*n* = 41 adolescents; *n* = 31 adults). Adolescents averaged a score of 36.8 (7% morning type). Adults averaged a score of 38.8 (30% morning type).

Adolescent participants were assessed for pubertal stage according to the criteria of Tanner (1962). Assessments were conducted by a pediatric board-certified physician. Participants were categorized as either late pubertal (Tanner 4) or post-pubertal (Tanner 5). Table 1 includes sample sizes for each Tanner stage, as well as the distributions of biological sex and age in each Tanner Stage.

**Table 1:** Demographics by Tanner stage^a^ in the adolescent group.

	**Tanner 4**	**Tanner 5**
Total (N)	23	21
Age (years)	15.8 ± 1.0 (14.3–17.8)	16.5 ± 0.9 (14.8–17.8)
Biological sex (N)		
Female	11	12
Male	12	9

^*a*^Stage of puberty based on secondary sexual characteristics (pubic hair) assessed by a board-certified pediatric physician.

Adolescents completed the study during the summer months (June, July, and August) during summer break of the academic school year. To minimize photoperiod differences between age groups caused by seasonal changes, adult participation was also restricted to the summer months but extended to include May and September. Photoperiod ranged from 12.05 h to 15.23 h. Study recruitment was conducted using online advertisements, locally posted flyers, mailed postcards, and word of mouth.

The study was approved by Rush University Medical Center's Institutional Review Board (Ethical approval number: ORA#10012801) in accordance with the Declaration of Helsinski. Adult participants provided written consent for participation. Legal guardians of adolescent participants signed written consent forms and adolescent participants signed the same form to provide assent for participation. All participants were paid for participation in the study.

### Protocol

This protocol took place as part of a larger study to construct a phase response curve (PRC) to bright light in adolescents (Crowley and Eastman, 2017) and was the same protocol used to examine differences in circadian period between adolescents and adults (Crowley and Eastman, 2018).

**Figure 1:**
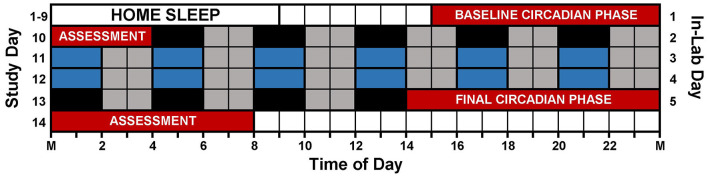
Study protocol. Prior to arriving to the lab, participants followed individualized, 9-h sleep schedules at home for 8 or 9 days. On in-lab day 1 (study day 10), participants completed a 13-h circadian phase assessment to determine baseline dim light melatonin onset (DLMO) starting at 15:00 (red). During the assessment, participants remained in very dim, red light (< 5 lux) and provided saliva samples every 30 min. Immediately following the phase assessment, participants began an ultradian light/dark protocol with 2 h of darkness (0 lux; depicted in black and blue) alternating with 2 h of dim room light (~20 lux; depicted in gray). Immediately following the ultradian LD protocol, participants completed a second circadian phase assessment. Only naps depicted in blue were included in the analysis (see text).

Figure 1 illustrates the study protocol. Prior to arriving for the laboratory session, participants followed a strict sleep schedule at home for 8 or 9 days. Participants were instructed to remain in the dark, in silence, without any distractions or technology usage for 9 h and attempt to sleep. Home sleep/wake schedules were assigned according to pre-study sleep patterns. Adolescent bedtimes ranged from 21:00 to 02:00, and adolescent wake times ranged from 06:00 to 11:00. Adult bedtimes ranged from 21:30 to 01:30, and adult wake times ranged from 06:30 to 10:30. Participants wore actigraphs (Actiwatch Spectrum, Philips Respironics, Bend, OR, USA) on their non-dominant wrist to measure compliance to the at home prescribed sleep schedule and to measure sleep in the lab. Participants completed sleep diaries to record detailed questions about his or her sleep such as bedtime, try to sleep time, number of wakings throughout night, time spent awake throughout night, wake time, lights on time, and out of bed time. Participants called an automated voicemail box twice a day, once at bedtime and once at wake. During the home sleep period, participants visited the lab every 2–3 days to download their actigraphy data. The information provided within the sleep diaries was reviewed and compared to the actigraphy data during the download appointments, and any inconsistencies were questioned by research staff.

After the home sleep period, participants lived in the lab for 5 days. They completed a circadian phase assessment at the beginning of the laboratory session to measure the dim light melatonin onset (DLMO), the most reliable marker of the circadian timing system (Klerman et al., 2002). Participants arrived at 12:00 (in-lab day 1), and the circadian phase assessment began at 15:00. Sampling began 30 min later, at 15:30, and samples were collected every 30 min for 13 h. For every sample, 2 mL of saliva were collected using Salivettes (Sarstedt, Nümbrecht, Germany). Samples were immediately centrifuged and then frozen and stored at −20°F. During saliva collection, participants sat reclined, in a comfortable chair in dim light (< 5 lux) and were asked to remain in their chair except for bathroom trips. For the 10 min preceding every sample, participants did not eat or drink and were not allowed to use the bathroom. The frozen samples were sent for analysis to SolidPhase, Inc. (Portland, ME, USA). There, commercial radioimmunoassays (Alpro, Salem NH, USA) were used to measure the salivary melatonin concentration of each sample. The intra-assay coefficient of variation was 4.1% for samples with low melatonin concentrations and 4.8% for samples with high melatonin concentration. The inter-assay coefficient of variation was 6.6% for samples with low melatonin concentrations and 8.4% for samples with high melatonin concentrations.

Participants followed an ultradian light/dark (LD) schedule for a total of 3.4 days. This protocol began immediately following the circadian phase assessment in a windowless room absent of any time cues such as clocks and devices with access to the internet. During this time, light (23.9 ± 7.1 lux) and dark (0 lux) periods cycled every 2 h (LD 2:2) to create a series of 4-h days. Overhead room lighting was produced from three fluorescent (4,100K) ceiling fixtures. Based on the standard FL11 and illuminance of 23.9 lux, we estimate that the melanopic equivalent daylight (D65) illuminance (EDI) was 13.43 lux (CIE S 026 α-opic Toolbox - v1.049a - 2020/11). During the dark periods (naps), participants were separated from each other with divider walls, noise disruptions were minimized using white noise machines, and participants were instructed to try to sleep in portable beds. Dark periods were immediately followed by light periods, during which, participants were allowed to eat freely, socialize with other participants, play games, read, and participate in other quiet activities.

Throughout the duration of the study, participants were prohibited from using medications that influence sleep such as anti-histamines, medications that influence melatonin such as non-steroidal anti-inflammatory drugs, recreational drugs such as marijuana, and nicotine. Urine panels were administered prior to study enrollment in order to confirm that participants had not consumed recreational drugs or nicotine. Alcohol was prohibited in the 5 days prior to the lab week. Breathalyzer tests were administered upon arrival to each laboratory session to confirm that participants had not consumed alcohol. Finally, participants were instructed to abstain from caffeine in the 3 days prior to the lab week and caffeine was prohibited throughout the in-lab session.

### Analysis

The DLMO was defined as the time when melatonin concentrations exceeded and stayed above a threshold of 4 pg/mL (Carskadon et al., 1997). The exact DLMO time was estimated using linear interpolation across the times at which the sample concentration bracketed the 4 pg/mL threshold (Carskadon et al., 1997; Crowley et al., 2016). Based on visual inspection of the melatonin profiles, thresholds were adjusted to 10 pg/mL (*n* = 2 adolescents) or 1 pg/mL (*n* = 1 adolescent) due to high or low melatonin production levels, respectively.

Each nap was scored using the sleep epochs sleep interval detection algorithm and the low wake threshold in Actiware 6 (version 6.0.9, Philips Respironics, Inc., Murrysville, PA, USA). The time at which participants attempted to fall asleep (rest interval start time) was set as the time that the lights were turned off for the nap in the laboratory by study staff. Sleep onset was defined as the first minute of 3 consecutive minutes of inactivity after lights out (Acebo et al., 1999). Wake time was defined as the first epoch of activity following 5 consecutive epochs of inactivity before lights on (rest interval end time). Two main outcomes were analyzed to assess sleep propensity across 24 h: sleep onset latency and total sleep time. Sleep onset latency was defined as the number of minutes between lights out time and sleep onset time. Total sleep time was the total amount of time scored as sleep between sleep onset and wake time.

**Figure 2:**
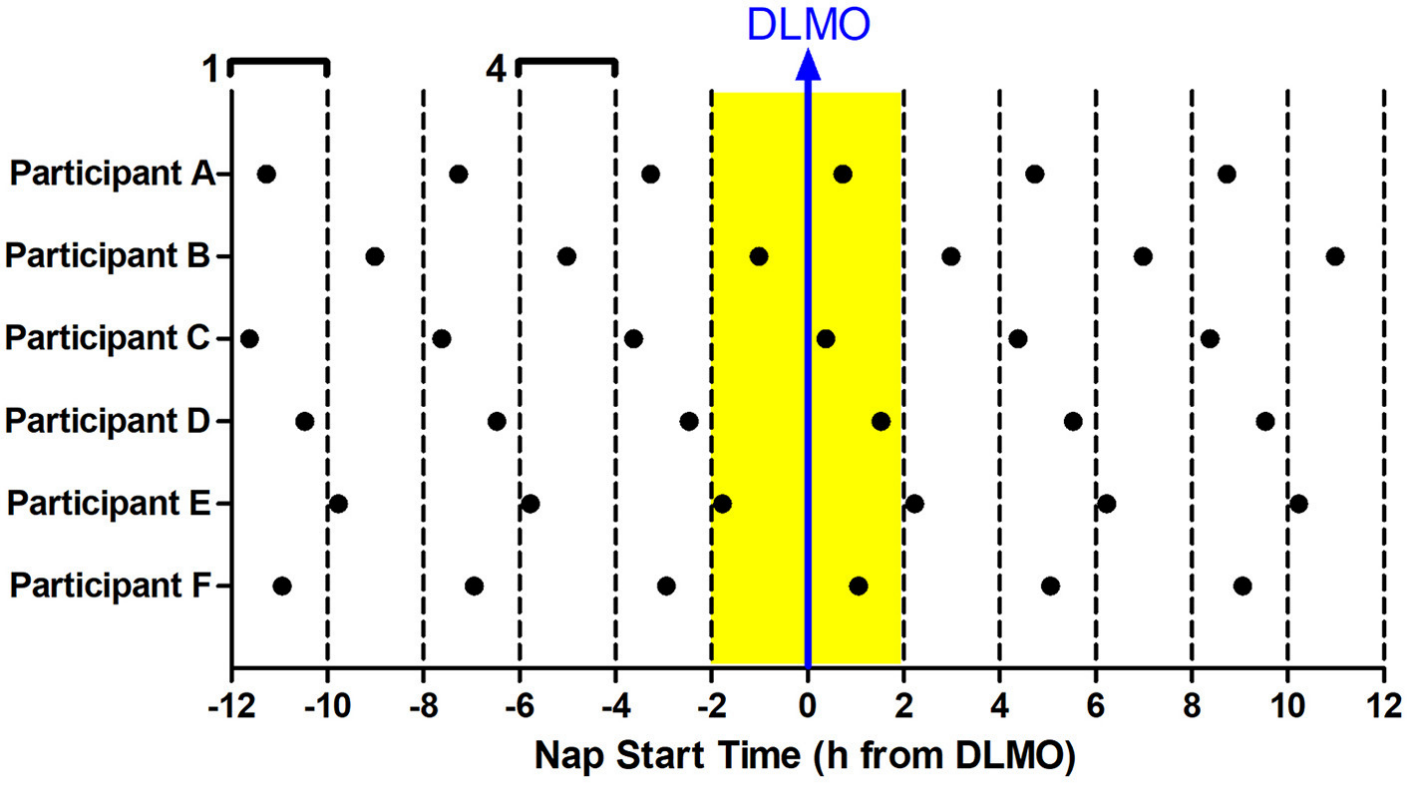
Binning of nap times relative to baseline DLMO for six example participants **(A–F)**. 12 naps were analyzed for each subject (in-lab days 3–4). Each nap began at the same time every day. Naps were analyzed relative to the individual's baseline DLMO. This analysis produced 6 nap timings for each subject, with two data points for each timing. These naps were then binned according to when they started with respect to baseline DLMO in 2-h windows. For example, any naps that started 10–12 h before the baseline DLMO were averaged for analysis (bin 1). Likewise, any naps that started 4–6 h before the baseline DLMO were averaged for analysis (bin 4). This produced 12 bins, each bin containing 28–56 naps. The midpoint of the bin is plotted on the x-axis in all graphs presented. The baseline DLMO is depicted by the blue arrow. The time slots designated as the forbidden zone for sleep for this analysis are indicated in yellow (bins 6 and 7).

The 12 Naps that occurred after the first 5 naps were chosen for analysis (naps 6–17; depicted in blue in Figure 1), which allowed for two naps to contribute to each time of day for each participant and allowed for some recovery after staying awake late for the baseline circadian phase assessment. Naps were then binned into 2-h windows based on when the nap opportunity began (lights out time) relative to baseline DLMO (see Figure 2). Each bin contained between 28 and 56 naps. Sleep did not occur for 34 naps (3.2% of total analyzed). These naps were not included in the sleep onset latency analysis due to failure to achieve sleep onset. For the total sleep time analysis, these naps were included and coded as 0 min. Previous work (Lavie, 1986; Strogatz et al., 1987; Dijk and Czeisler, 1994, 1995) shows that the forbidden zone for sleep occurs in the 2–3 h before habitual bedtime, which also overlaps with the time of the dim light melatonin onset. Therefore, we defined the 2 h before and 2 h after the DLMO as the forbidden zone (yellow highlight in Figure 2).

Two-level mixed linear models with naps nested within individuals were used to examine age (adolescent vs. adult) and sex differences in sleep propensity for the naps within the defined forbidden zone (±2 h from DLMO). The level 1 models predicted total sleep time or sleep onset latency from time of day (whether the nap started during the defined forbidden zone or not), age (adolescent or adult), biological sex, and their interactions. The level 2 models estimated average effects across individuals for the level 1 predictors, plus individual-specific deviations in the intercepts. Based on these results, age and sex differences in sleep propensity outcomes were examined for naps that started within the forbidden zone (± 2 h of DLMO) *post-hoc* using unpaired *t*-tests. Group difference effect sizes (Cohen's d) are also reported.

## Results

The mean DLMO for adolescents was 21:43 ± 1:35 (minimum = 19:07; maximum = 1:09). The mean DLMO for adults was 21:29 ± 1:31 (minimum = 18:46; maximum = 1:11). Both adolescents and adults received over 8 h of sleep per 24-h period (adolescent mea*n* = 8.2 ± 1.4 h, adult mea*n* = 8.7 ± 1.5 h).

**Figure 3:**
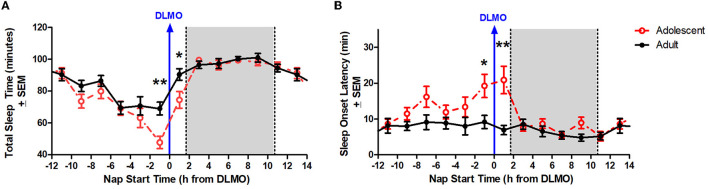
Age differences in total sleep time **(A)** and sleep onset latency **(B)** across the circadian day. The red lines illustrate adolescent sleep outcomes for each 2-h bin relative to their DLMO (blue line at 0). The black line illustrates adult sleep outcomes. Means are plotted at the midpoint of the 2-h bin. The gray box represents the average time of habitual sleep relative to DLMO. Differences between the age groups were analyzed for naps that started within the forbidden zone for sleep (2-h bin before and 2-h bin after DLMO) using unpaired t-tests. Significance is indicated by asterisks (*p < 0.05; **p < 0.005).

Figure 3A illustrates total sleep time for adolescents and adults across 24 h and relative to their DLMO. The mixed model revealed a significant main effect of the interaction between time of day and age for total sleep time (*b* = −16.2, *p* = 0.01) with adolescents receiving significantly less total sleep than adults during the forbidden zone. Compared to adults, adolescents slept less in the 2 h window before their DLMO [47.9 ± 29.3 min vs. 69.1 ± 28.5 min; *t*_(51)_ = −2.91, *p* = 0.01; *d* = 0.81] and in the 2 h after their DLMO [74.5 ± 30.1 min vs. 90.5 ± 22.5 min; *t*_(33)_ = −1.99, *p* = 0.05; *d* = 0.67].

Figure 3B illustrates sleep onset latency for adolescents and adults across 24 h and relative to their baseline DLMO. The mixed model revealed a significant main effect of the interaction between time of day and age for sleep onset latency (*b* = 12.8, *p* < 0.001) with adolescents exhibiting significantly longer sleep onset latency than adults. Compared to adults, adolescents took longer to fall asleep in the 2 h window before their DLMO [19.2 ± 24.1 vs. 9.2 ± 13.1; *t*_(50)_ = 2.13, *p* = 0.04; *d* = −0.60] and in the 2 h after their DLMO [20.9 ± 21.6 vs. 7.0 ± 7.8; *t*_(33)_ = 3.25, *p* = 0.003; *d* = −1.07].

**Figure 4:**
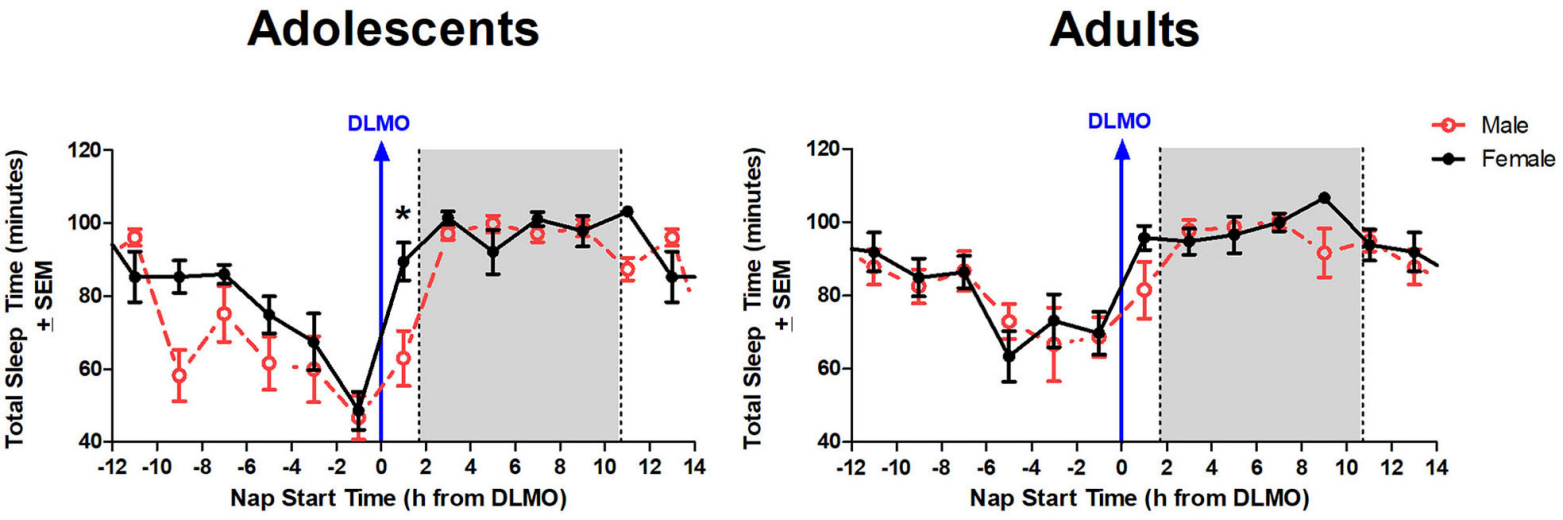
Sex differences in total sleep time across the circadian day. The red line indicates average male total sleep time for each 2-h bin relative to their DLMO for each age group. The black line represents average female total sleep time for each 2-h bin relative to their DLMO for each age group. The gray box represents the average time of the habitual sleep period relative to baseline DLMO. Differences between biological sexes were analyzed for naps that started within the forbidden zone for sleep (2-h bin before and 2-h bin after DLMO) using unpaired *t*-tests. Significance is indicated by asterisks (*p < 0.05).

The mixed model revealed a significant main effect of the interaction between time of day and sex for total sleep time (*b* = −12.3, *p* = 0.03). Further analysis revealed that male adolescents slept less than female adolescents during the 2 h window after the DLMO [*t*_(14)_ = −2.24, *p* = 0.04; *d* = 1.18]. On average, adolescent males slept 62.9 ± 32.2 min compared to adolescent females who slept 89.4 ± 19.4 min, on average (Figure 4). A similar trend was observed within the adult age group; however, the difference did not reach statistical significance [*t*_(17)_ = −1.47, *p* = 0.16; *d* = 0.63].

**Figure 5:**
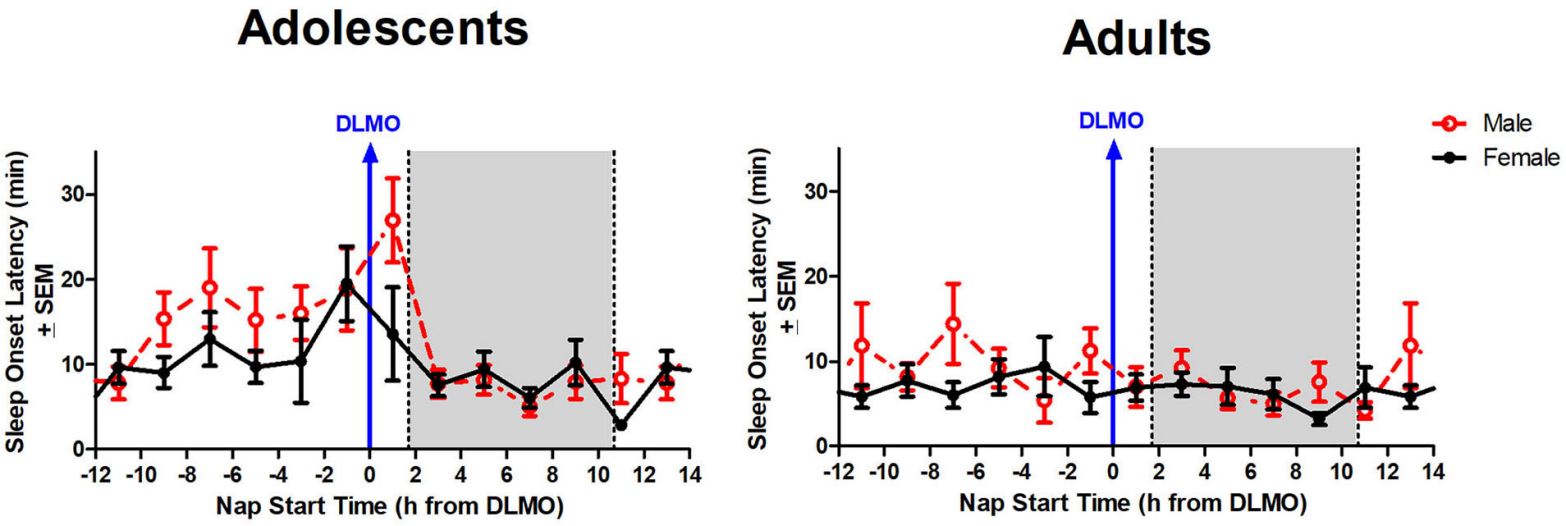
Sex differences in sleep onset latency across the circadian day. The red line indicates average male sleep onset latency for each 2-h bin relative to their DLMO for each age group. The black line represents average female sleep onset latency for each 2-h bin relative to their DLMO for each age group. The gray box represents the average time of habitual sleep relative to baseline DLMO. Differences between biological sexes were analyzed for naps that started within the forbidden zone for sleep (2-h bin before and 2-h bin after DLMO) using unpaired *t-*tests; no sex differences were detected.

The interaction between time of day and sex for sleep onset latency was non-significant (*b* = 4.3, *p* = 0.10), though results trended in the direction of a longer sleep onset latency for males compared to females when naps started during the forbidden zone. Figure 5 illustrates that the adolescent male group took longer to fall asleep than the adolescent females in the 2 h after the DLMO [*t*_(14)_ = 1.81, *p* = 0.09; *d* = −0.94].

To explore whether being late or post-pubertal may be driving heightened arousal around the time of the DLMO in adolescents, we compared total sleep times and sleep onset latencies between Tanner 4 and Tanner 5 participants. Total sleep times 2 h before the DLMO [*t*_(26)_ = 1.55, *p* = 0.13] and 2 h after the DLMO[*t*_(14)_ = −1.80, *p* = 0.93] did not differ between Tanner stages 4 and 5. Similarly, sleep onset latencies 2 h before the DLMO [*t*_(25)_ = 0.45, *p* = 0.66] and 2 h after the DLMO [*t*_(14)_ = 1.61, *p* = 0.13] did not differ between Tanner stages 4 and 5.

## Discussion

The current analysis aimed to examine the circadian-driven alertness signal – the so-called “wake- maintenance zone” or “forbidden zone for sleep” – of late/post-pubertal adolescents to determine whether this may be an additional mechanism contributing to delayed nocturnal sleep propensity commonly reported in this age group. Using a 4-h day protocol to minimize homeostatic sleep pressure, our data revealed that adolescents have more difficulty initiating and maintaining sleep during naps timed within 2 h of the dim light melatonin onset (DLMO) compared to adults. This increased arousal was observed despite adolescents being in a time-free environment with no access to phones, tablets, or other external influences that may enhance alertness in the evening before bedtime. These findings suggest that a robust forbidden zone for sleep (i.e., greater amplitude of the sleep propensity rhythms in the hours around DLMO) may be an additional mechanism that contributes to delayed sleep onset observed in post- pubertal adolescents.

The Two-Process Model of Sleep Regulation (Borbély, 1982; Carskadon and Acebo, 2002; Carskadon et al., 2004; Jenni and LeBourgeois, 2006) depicts a homeostatically-regulated sleep/wake system (Process S) and an ~24-h circadian clock (Process C) ideally working together to consolidate sleep and wakefulness across the day. Process S is use-dependent and defined by the accumulation of sleep propensity across waking and dissipation across a night of sleep. The circadian rhythm of sleep propensity also fluctuates across the day with a period of ~24 h. When aligned, Process C promotes the greatest sleep propensity toward the end of habitual sleep and therefore maintains sleep upon the dissipation of Process S and Process C promotes alertness in the evening as wake-dependent sleep pressure builds toward the end of the day. During adolescence, the circadian timing system shifts later (phase delay), and the buildup of sleep pressure during waking slows (Carskadon et al., 1997; Jenni et al., 2005; Taylor et al., 2005). These changes contribute to enhanced evening alertness and are thought to be permissive of later bedtimes and delayed sleep onset as adolescents mature. The findings from the current analysis, however, suggest that the strength or amplitude of the circadian-dependent alerting at the end of the waking day is another physiological factor that may contribute to late sleep onset in post-pubertal adolescents. We propose that all three factors – a slow accumulation of sleep pressure across waking, a delayed circadian phase, and a robust amplitude in the circadian-driven forbidden zone for sleep – contribute to early sleep onset difficulties in post-pubertal adolescents.

Although less studied than circadian phase, circadian amplitude – the difference between the peak (or trough) and the mean of a ~24-h rhythm – has been described previously in the context of age-related reductions in the sleep/wake, melatonin, and core body temperature rhythms in adults (Czeisler et al., 1992; Monk et al., 1995; Duffy et al., 1998; Dijk et al., 2000; Münch et al., 2005). Previous modeling work has simulated the impact of central circadian pacemaker amplitude changes on sleep timing across the lifespan (Phillips et al., 2010; Skeldon et al., 2015). These models demonstrate that circadian amplitude and sleep timing have a positive correlation, with increased circadian amplitude corresponding to later sleep times and decreased circadian amplitude corresponding to earlier sleep timing and morningness. Based on these simulations, the researchers concluded that the late sleep timing of adolescents can be partly explained by a greater circadian amplitude and that the shift to earlier sleep across the lifespan is likely associated with a reduction in circadian amplitude. The findings from the present analysis are consistent with these previous models. The circadian rhythm of sleep propensity as demonstrated by sleep onset latency and total sleep time across the 24-h day demonstrate an increased circadian amplitude in these variables for adolescents compared to adults aged 30–45 years (see Figure 3).

Previous research has investigated sleep propensity across the circadian day through the use of ultradian protocols similar to that of the present study. Münch et al. (2005) conducted a 4- day protocol with a 225-min day; periods of 75 min of sleep alternated with 150 min of wake. Similar to the present analysis, this protocol compared sleep propensity in an older and younger population; however, they focused exclusively on adults, with the younger population consisting of emerging and young adults aged 20–31 years and the older population consisting of older adults aged 57–74 years. This analysis also revealed an age-related difference in sleep propensity in the region of the forbidden zone for sleep when controlling for individual DLMO, with younger adults exhibiting decreased ability to fall asleep (sleep onset latency) and maintain sleep (total sleep time). When considering these findings together with the current findings, the results provide an understanding of sleep propensity across the life span beginning in post-pubertal adolescence and ending in older adulthood and suggests that the robust forbidden zone for sleep observed in post-pubertal adolescence persists into emerging and early adulthood but progressively dissipates with age. Analyses including all stages of life, including earlier development stages such as pre-pubertal children, are warranted in order to determine when the forbidden zone for sleep is most robust and the rate at which amplitude declines across the lifespan.

In addition to the age-based effects on sleep propensity around the time of the DLMO, a sex difference also emerged. This difference appears to be driven by adolescent males, as this group displayed a significant decrease in total sleep time in naps beginning in the 2 h after the DLMO compared to adolescent females, while the adult age group did not display a significant sex difference. Biological sex differences and sleep propensity during the forbidden zone for sleep were previously investigated in our lab (Eastman et al., 2017) in 49 young adult participants aged 18–44 years, who participated in a similar time-free ultradian protocol, alternating between 2 h nap opportunities and 3 h periods of wake. In this analysis, the forbidden zone for sleep also occurred around the time of the DLMO, but no sex differences emerged. Taking the current data together with this previous work may suggest that this sex difference dissipates with age. More likely, however, is that adolescent boys have more motor activity across the sleep period than girls (Short et al., 2012; Meltzer et al., 2019). Therefore, this sex difference needs to be confirmed with a non-activity based measurement of sleep like polysomnography.

The impact of decreased sleep propensity in the evening described here has potential implications for strategies used to increase sleep duration in adolescents to the current sleep duration recommendations of 8 to 10 h per night (Hirshkowitz et al., 2015; Paruthi et al., 2016), particularly on school nights. The morning social clock of adolescents is primarily driven by school start times. Although delaying school start times has gained traction in many communities (Dunster et al., 2018; Meltzer et al., 2021; Ziporyn et al., 2022; Mousavi and Troxel, 2023), there remains a need to develop strategies for middle and high school students who have difficulty getting sufficient sleep during the school week. We and others have demonstrated positive effects of advancing habitual bedtime to extend sleep duration (Beebe et al., 2013, 2017; Van Dyk et al., 2017; Crowley et al., 2023), including improved mood and focus, as well as healthy food choices. These benefits, however, are not experienced by all adolescents, especially those with a late chronotype (Beebe et al., 2015). Moreover, while advancing bedtime allows for increased total sleep duration, adolescents are more likely to have a harder time falling asleep. Campbell et al. (2023) recently reported on data examining the effects of increasing sleep duration by advancing bedtime in adolescents. For up to 3 years, a younger cohort (~10–14 years) completed 3 sleep duration conditions (7 h, 8.5 h, and 10 h) for four consecutive nights each in which bedtime was shifted earlier (advanced). Another cohort (15–20 years) completed the same conditions once. With an increase in time-in-bed, sleep duration increased. Sleep onset latency, however, also increased with earlier bedtimes, averaging 20 min or more in the 10 h time-in-bed condition across age groups. An age-dependent increase in sleep onset latency also emerged in all conditions; by about 18 years, sleep onset latency averaged ~10 min in the 7 h condition, ~15 min in the 8.5 h condition and ~25 min in the 10 h time-in-bed condition. Focusing on the 10 h condition only, the authors reported that ~36% of the older quartile took more than 30 min to fall asleep and ~10% took more than 1 h to fall asleep. This is compared to less than 20% of the younger quartile taking more than 30 min to fall asleep and no one in this younger group taking more than 1 h to fall asleep when given a 10 h sleep opportunity. Based on the data from the current analysis, the lengthening of sleep onset latency with age in this previous study is likely a circadian-driven phenomenon (phase and amplitude); however, circadian outcomes were not measured.

We recommend that earlier bedtimes should be coupled with techniques to phase advance the circadian system. Advancing the circadian system would, in turn, advance the forbidden zone for sleep to an earlier time of day, decreasing the difficulty to fall asleep at the earlier target bedtimes. We recently reported data from a two-week intervention in which bedtime was gradually advanced (1 h during the first week and 2 h during the second week) in late- and short-sleeping adolescents (14–17 years) attending high school (Crowley et al., 2023). To facilitate these early bedtimes and the ability to fall asleep at these earlier times, we used two strategies. First, adolescents sat in front of a bright light box (~6000 lux; 2.5 h h) on both mornings of the weekend following week 1. The bright light was timed to begin at the most sensitive time to produce phase advance shifts in adolescents (Crowley and Eastman, 2017). Second, to account for the increasing societal pressures such as homework demands and more access to technology, we also used an individualized time management plan, “Sleep RouTeen” to facilitate earlier bedtimes and increase the feasibility of meeting those expectations. This 2-week intervention advanced the dim light melatonin onset (DLMO) by about 40 min on average, sleep onset advanced by 90 min, and sleep duration increased by 1.2 h (Crowley et al., 2023). Although sleep onset latency during the second week did not differ between adolescents in the intervention group compared to a control group, it increased from 8.5 to 20 min on average. Upon further investigation, this increase in sleep onset latency was partly driven by a few adolescents who did not advance their DLMO or showed very little change in phase. Therefore, difficulty falling asleep likely emerged because their circadian clock did not shift earlier, and we were asking these adolescents to try to fall asleep close to their forbidden zone for sleep.

There are some limitations to the current analysis. First, the analysis of sleep onset latency and total sleep time was limited to actigraphy and not polysomnography (PSG). While actigraphy has been validated as an accurate measure of sleep compared to PSG (De Souza et al., 2003; Meltzer et al., 2016) and lights out/try to fall asleep time were controlled by the laboratory, it is not as accurate as PSG, particularly with respect to measuring sleep onset latency. Adults in the current study show little circadian variation in sleep onset latency (Figure 3), yet previous work describes a clear circadian rhythm in sleep onset latency using PSG that tracks core body temperature (Dijk and Czeisler, 1994; Gradisar and Lack, 2004). Further investigation of the forbidden zone for sleep in adolescents using PSG is warranted in order to validate the findings described here. Another limitation of the present analysis is that this study was limited to late/post-pubertal adolescents. Thus, it is unclear whether the observed differences are limited to mature adolescents or if earlier stages of development experience a similar robust forbidden zone for sleep. Finally, this study was limited to only healthy adolescents and adults. Thus, it is unclear whether the same effects are observed in clinical populations such as individuals diagnosed with Delayed Sleep Wake Phase Disorder. Further investigations into this subgroup in particular are warranted due to the relevance of any modifications to clinical and behavioral recommendations for advancing circadian phase and sleep in this clinical population.

The present analysis demonstrates that healthy late to post-pubertal adolescents display a more robust forbidden zone for sleep compared to adults aged 30–45 years. These findings suggest that adolescents display a more pronounced circadian-dependent evening alertness signal than adults. We propose that heightened evening alertness experienced by mature adolescents may be caused by a combination of a delayed circadian phase, slowed buildup of homeostatic sleep pressure during waking, and an increase in circadian amplitude. Given the increased difficulty in falling asleep in the 4 h preceding habitual bedtime, recommendations for shifting bedtimes earlier in adolescents must implement circadian-based strategies in order to make these changes more feasible for adolescents.

## Data availability statement

The original contributions presented in the study are included in the Supplementary material; further inquiries can be directed to the corresponding author.

## Ethics statement

The studies involving humans were approved by the Rush University Medical Center Institutional Review Board #1. The studies were conducted in accordance with the local legislation and institutional requirements. Written informed consent for participation in this study was provided by the participants' legal guardians.

## Author contributions

AM: Data curation, Visualization, Writing—original draft. JA: Formal analysis, Writing—review & editing. CE: Writing—review & editing, Conceptualization, Methodology. SC: Conceptualization, Methodology, Writing—review & editing, Data curation, Funding acquisition, Investigation, Project administration, Resources, Supervision, Visualization.
